# A practical guide to necropsy of the elasmobranch chondrocranium and causes of mortality in wild and aquarium-housed California elasmobranchs

**DOI:** 10.3389/fvets.2024.1410332

**Published:** 2024-06-13

**Authors:** Ri K. Chang, Mark S. Okihiro

**Affiliations:** ^1^Monterey Bay Aquarium, Monterey, CA, United States; ^2^California Department of Fish and Wildlife, Vista, CA, United States

**Keywords:** elasmobranch, necropsy, stranding, chondrocranium, *Miamiensis avidus*, *Carnobacterium maltaromaticum*, meningoencephalitis, otitis

## Abstract

Elasmobranchs are common, iconic species in public aquaria; their wild counterparts are key members of marine ecosystems. Post-mortem examination is a critical tool for disease monitoring of wild elasmobranchs and for management of those under human care. Careful necropsy of the head, with a focus on clinically relevant anatomy, can ensure that proper samples are collected, increasing the chance of presumptive diagnoses prior to slower diagnostic workup. Immediate feedback from a thorough head necropsy allows for faster management decisions, often identifying pathogens, routes of pathogen entry, and pathogenesis, which are current shortcomings in published literature. This article proposes a protocol for necropsy of the elasmobranch chondrocranium, emphasizing unique anatomy and careful dissection, evaluation, and sampling of the endolymphatic pores and ducts, inner ears, brain, and olfactory system as part of a complete, whole-body necropsy. Extensive use of cytology and microbiology, along with thorough sample collection for histology and molecular biology, has proven effective in identifying a wide range of pathogens and assisting with characterization of pathogenesis. The cause of mortality is often identified from a head necropsy alone, but does not replace a thorough whole-body dissection. This protocol for necropsy and ancillary diagnostic sample collection and evaluation was developed and implemented in the necropsy of 189 wild and aquarium-housed elasmobranchs across 18 species over 13 years (2011–2023) in California. Using this chondrocranial approach, meningoencephalitis was determined to be the primary cause of mortality in 70% (118/168) of stranded wild and aquarium-housed elasmobranchs. Etiology was largely bacterial or protozoal. *Carnobacterium maltaromaticum* bacterial meningoencephalitis occurred in salmon sharks (*Lamna ditropis*), shortfin mako sharks (*Isurus oxyrinchus*), common thresher sharks (*Alopias vulpinus*), and one Pacific electric ray (*Tetronarce californica*). *Miamiensis avidus* was the most common cause of protozoal meningoencephalitis and found almost exclusively in leopard sharks (*Triakis semifasciata*) and bat rays (*Myliobatis californica*) that stranded in San Francisco Bay. Bacterial pathogens were found to use an endolymphatic route of entry, while protozoa entered via the nares and olfactory lamellae. Trauma was the second most common cause of mortality and responsible for 14% (24/168) of wild shark strandings and deaths of aquarium-housed animals.

## Introduction

Post-mortem examination is a critical tool for disease monitoring of wild, stranded elasmobranchs and management of sharks, rays, and skates under human care. Necropsies performed with a thorough understanding of taxa-specific anatomy can ensure that appropriate samples are collected, increasing the chance of presumptive diagnoses prior to slower diagnostic workup (e.g., histology, microbiology, and molecular biology), and allowing for faster management decisions. Wildlife necropsy is also a critical tool for disease surveillance and building our anatomic knowledge for understudied species (1, 2).

Elasmobranchs are a common taxa in aquaria and their wild counterparts are key members of their ecosystems (3). Infectious and inflammatory diseases are the most common cause of mortality of aquarium-housed elasmobranchs, with the gastrointestinal tract, gills, and brain being the most commonly affected sites (4, 5). Infectious meningoencephalitis has been reported in wild, stranded elasmobranchs, notably by the scuticociliate protozoa, *Miamiensis avidus*, and Gram-positive bacterium, *Carnobacterium maltaromaticum*, which have caused epizootic and enzootic strandings (6–8). *Miamiensis avidus* and the closely related *Philasterides dicentarchi* have also been implicated in deaths of aquarium-housed elasmobranchs (9–12). Potential routes of entry for pathogens include the endolymphatic ducts, olfactory system, ampullary system, and hematogenous spread, but are often undetermined in the current literature (8–11, 13–15).

This article proposes a protocol for the necropsy of the elasmobranch chondrocranium, emphasizing careful dissection of the endolymphatic pores/ducts, inner ear, brain, and olfactory system, providing methods for gross necropsy, sample collection for microbiology, cytology, molecular biology, and histopathology, and evaluation of wet-mount and stained cytology. The proposed protocol for necropsy and ancillary diagnostic sample collection and evaluation was developed through work with stranded, wild and aquarium-housed elasmobranchs and has been implemented in the necropsy of over 180 sharks and rays across 18 species from 2011–2023. The aim of this article is to highlight clinically relevant anatomy of the elasmobranch chondrocranium and offer guidance to those performing necropsies of animals both under managed care and free ranging.

## Materials and equipment

- *General necropsy instruments* – exact tools will depend on personal preference, but these authors have found Russian tissue forceps or rat-tooth forceps, Brown-Adson tissue forceps, Miltex dressing forceps, Mayo blunt/blunt scissors, iris scissors, and a #4 scalpel handle to work well. A large supply of sterile disposable #20 scalpel blades is necessary as blades will dull quickly when cutting skin and placoid scales. A #3 scalpel handle and #10 blades may be sufficient for smaller sharks and elasmobranchs without placoid scales. A heavy, fixed blade knife also has utility for some larger animals. A tissue spatula or other flat to slightly curved instrument for removing the brain is helpful. Disinfection materials, such as Clorox^™^ disinfecting wipes or 70% isopropyl alcohol on gauze, are used to minimize external contaminants prior to culture. General necropsy personal protective equipment, including eye protection, is a must.
- *Fluid sampling* – these tools are used for the sterile collection of fluids for cytologic, molecular biologic (e.g., PCR and metagenomic next generation sequencing) and microbiologic analysis. Sterile disposable transfer pipettes (1 and 5 mL), with tips small enough to enter the inner ear or sterile syringes (0.5, 1, or 3 mL) and needles (20, 22, or 25 gauge) are essential. Subcutaneous tissues surrounding endolymphatic ducts may not hold enough fluid to be easily collected and a cotton-tipped applicator may be useful in these instances. One mL cryovials are needed to store, freeze, and ship molecular biology samples.
- *Cytology and light microscopy* – a high quality binocular light microscope, with 4, 10, 20, and 40× plan achromatic dry objectives, is recommended. An ocular micrometer (to measure pathogen size), 60× dry, and 100× oil immersion objectives are also desirable. Dark-field condensers are extremely useful for identifying host cells and microorganisms in wet-mount cytology preparations. If a dark-field condenser is unavailable, the standard microscope condenser can be lowered to help visualize pathogens and host cells. Phase contrast may also highlight cells and pathogens, but is often less useful and more confusing than dark-field. Cytology samples are prepared on conventional 25 × 75 mm glass slides with glass coverslips.
- *Wright-Giemsa or modified Wright-Giemsa stains* (e.g., Dip Quick Stain by Jorgensen Laboratories, Stain Dip Quick by Covetrus, RAL Diff-Quik^™^ by Siemens, or HEMA 3^®^ by Protocol/Fisher Scientific) – although not strictly necessary, staining air-dried slides will help identify host cell types, better visualize bacteria and other microorganisms, and allow slides to be archived.
- *Bacterial and fungal culture* – there are many ways of identifying microbiologic agents. The most inexpensive is to prepare agar plates in-house and submit plates with growth for identification. Tryptic soy agar with 5% sheep red blood cells (blood agar) is typically used for bacterial pathogens and Sabouraud-Dextrose (Sab-Dex) agar for suspect fungal pathogens (16, 17). Blood agar and Sab-Dex are widely available commercially, at minimal expense. For most elasmobranch necropsies, both Sab-Dex and blood agar are used. While plate incubation at the appropriate ambient (i.e., ocean or display enclosure) water temperature is ideal, culture at room temperature has proven adequate for commonly encountered elasmobranch pathogens in these authors’ experience. Although colony morphology, combined with cytology, can often be used to classify and characterize pathogens, isolates are usually sent to outside diagnostic labs for precise identification. Diagnostic labs typically use matrix assisted laser desorption ionization-time of flight mass spectrometry (MALDI-TOF), 16S rRNA PCR, or conventional biochemical identification methods to determine pathogen genus and species.
- *Histology* – submission of histology samples to a laboratory well-versed in elasmobranch pathology will help to define normal, confirm gross and cytological findings, and provide additional details on pathogens, pathology, and pathogenesis. Supplies include 10% neutral buffered formalin, leakproof containers, and histology cassettes.
- *Photography equipment* – although digital single-lens reflex (DSLR) cameras may show the greatest detail, with adequate lighting and sample preparation, cell phone cameras and inexpensive point-and-shoot cameras (e.g., Sony Cybershot) are more than sufficient for documenting findings. Small lightweight compact cameras can be used one-handed and are often preferable to heavy DSLRs when the subject matter requires dynamic manipulation (e.g., reflecting the skin or calvarium) to expose a lesion or display a specific anatomic feature. This way manipulation can be done with one hand while taking photos with the other. Emphasis should be on taking high quality photos by (1) clearing the field of interest of debris, (2) blotting dry any pooled liquid (e.g., blood or extradural fluid), (3) using white paper or poster board as a nonreflective matte background, and (4) using diffuse indirect lighting (e.g., avoiding direct sunlight and artificial spotlights). Photos should be taken with and without a metric scale bar, so that the size of lesions and anatomy can be determined. Regardless of ambient lighting, photos should be taken with and without flash, to ensure proper exposure.

## Methods

### Specimen collection and preparation

Ideally specimens are necropsied as fresh as possible. If there is less than a 72 h delay, keeping the carcass on ice or in a walk-in cold room, but not frozen, is recommended. A longer delay will lead to significant autolysis. If immediate necropsy is not possible, consider freezing the specimen. Although freezing may preclude histopathology interpretation, diagnoses based on cytology, molecular techniques, and microbiology may still be possible.

Prior to necropsy, the animal should be thoroughly cleaned. This is especially important with stranded animals that are often covered by sand, mud, and debris. Spraying with clean saltwater or tap water will remove extraneous material from external skin surfaces, nares, the oral cavity, and gills. Cleaning the animal allows wounds to be accurately assessed and for normal anatomic features to be identified and photographed.

Complete morphometric data should be taken. For sharks, this includes body weight, total and fork lengths, and girth at a minimum. For rays, body weight, total length, disc length and width should be determined. External lesions and parasites should be counted, described, and parasites preserved in 95% ethanol.

Elasmobranch species should be determined by examination of (1) overall body size and shape, (2) dentition, (3) size, shape, and location of fins, and (4) skin pigmentation. Should questions remain as to species identity, a DNA sample (i.e., piece of fin or muscle) can be taken and frozen or preserved in 75–90% ethanol. Determine sex by the presence (male) or absence (female) of claspers. Thorough use of photography during the necropsy is critical for documenting anatomy and lesions. Anatomy varies widely among elasmobranchs and documenting anatomy for each species will help better distinguish normal from abnormal in future necropsies.

### Overall necropsy approach

Although emphasis here is on careful dissection of the head and chondrocranium, this is not to imply that the rest of the animal should be ignored. Prioritization of the head is necessary because a full necropsy is time consuming, often requiring more than 8 h to complete, and evaluation of chondrocranium has proven to be diagnostic more often than not. To minimize autolysis, the head can be detached from the trunk and examined separately while the rest of the animal is iced or refrigerated.

Following completion of the chondrocranial dissection, the rest of the animal can be examined in depth. This would include assessment of the branchial cavity and gills, heart, and major coelomic cavity organs. Details of a complete elasmobranch necropsy are beyond the scope of this paper, but the extent of trunk, cardiac, and coelomic cavity exam are usually dictated by finding in the head. If no cause of morbidity and mortality is found in the head, then there is greater emphasis on careful necropsy of the remainder of the animal. In contrast, if a definitive cause of death is found in the head, then more time can be spent on documenting normal anatomy and ancillary lesions or parasites.

### Chondrocranial approach

**Figure 1:**
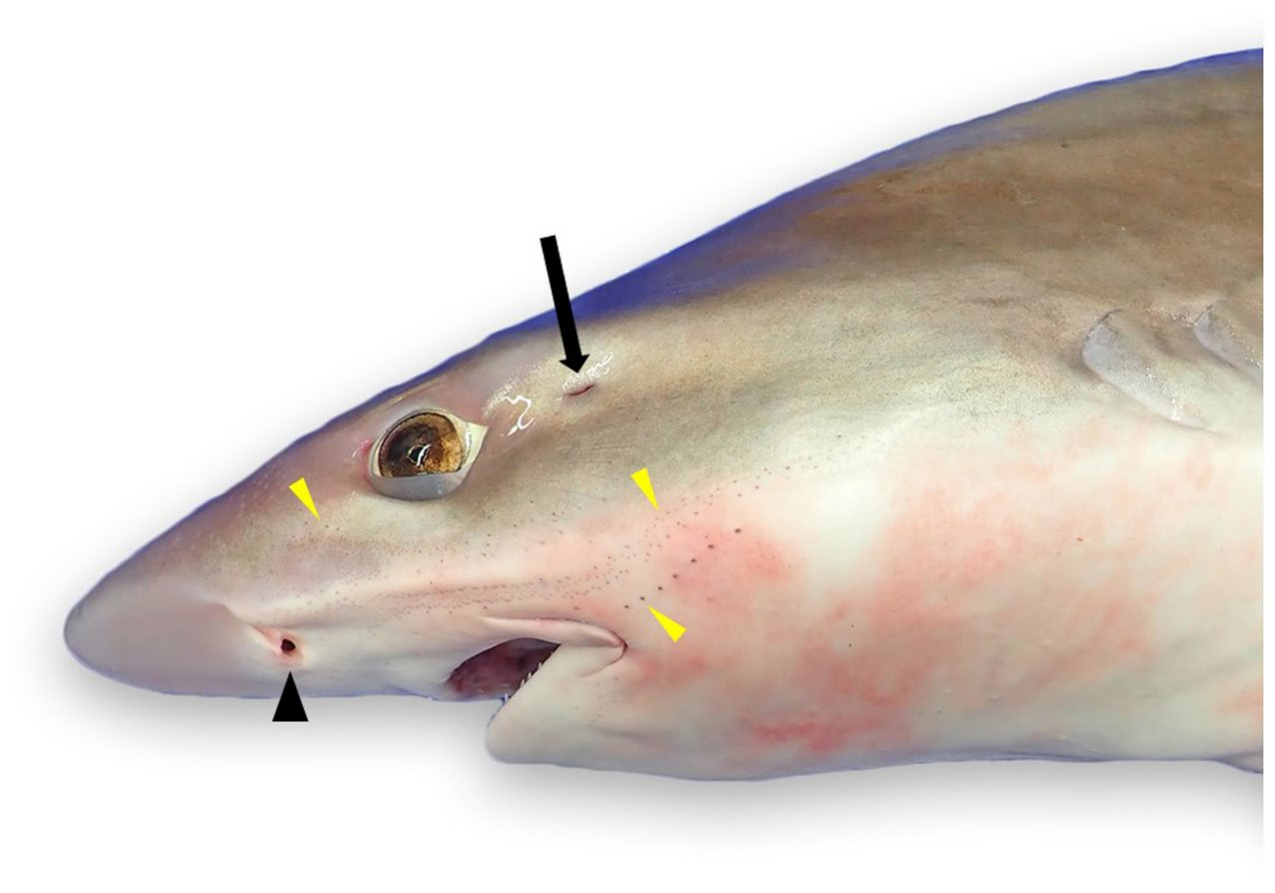
External landmarks for chondrocranial necropsy of a tope shark (*Galeorhinus galeus*): naris (black arrowhead), spiracle (black arrow), and numerous ampullae of Lorenzini and/or pores of the cephalic lateral line system (yellow arrowheads).

**Figure 2:**
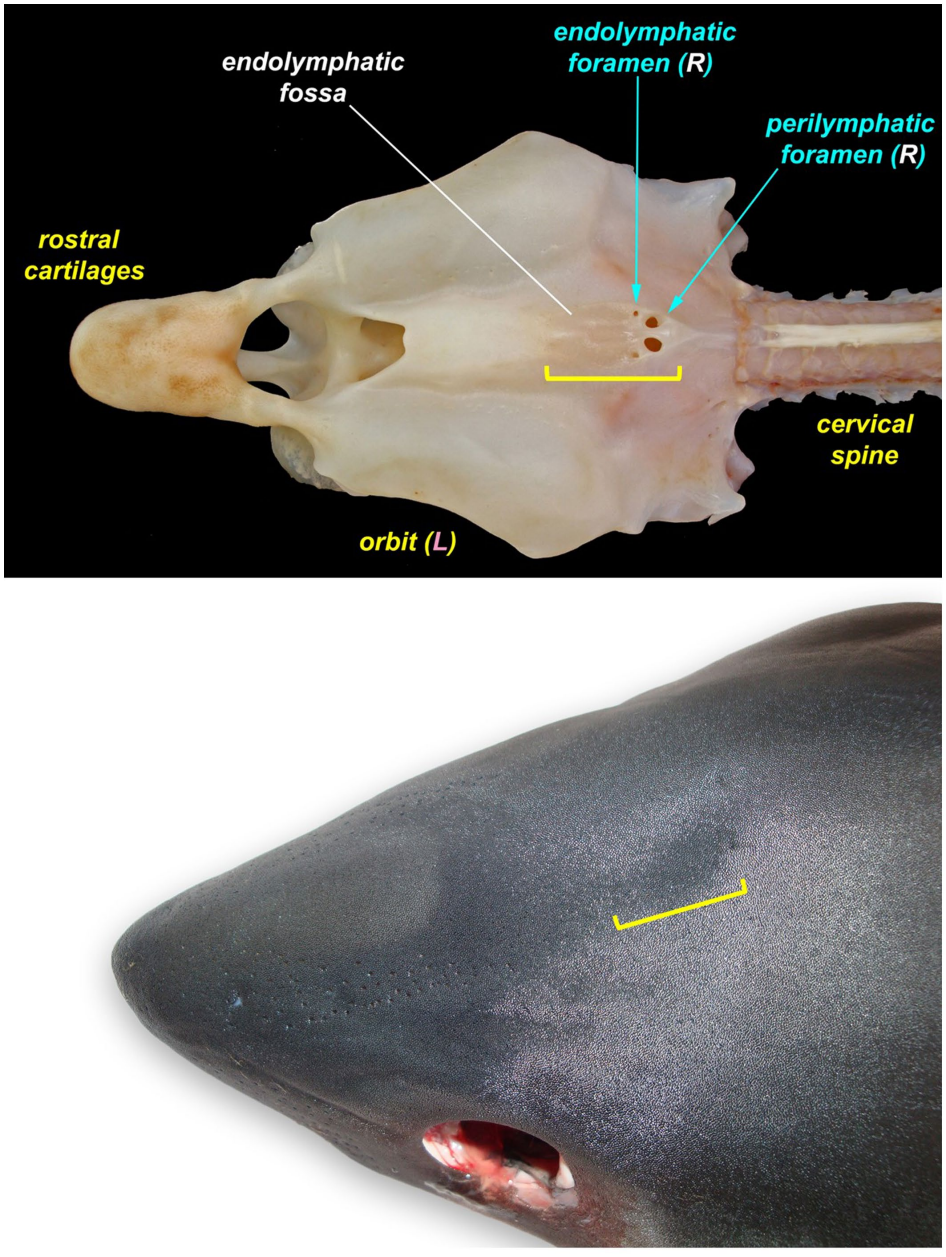
The endolymphatic fossa (yellow bracket) of a juvenile salmon shark (*Lamna ditropis*). The endolymphatic fossa is a palpable depression on the dorsal chondrocranium with endolymphatic pores located at the caudal margin. The fossa can be used to identify the location for the initial incision around the endolymphatic pores even when the pores are not visible.

**Figure 3:**
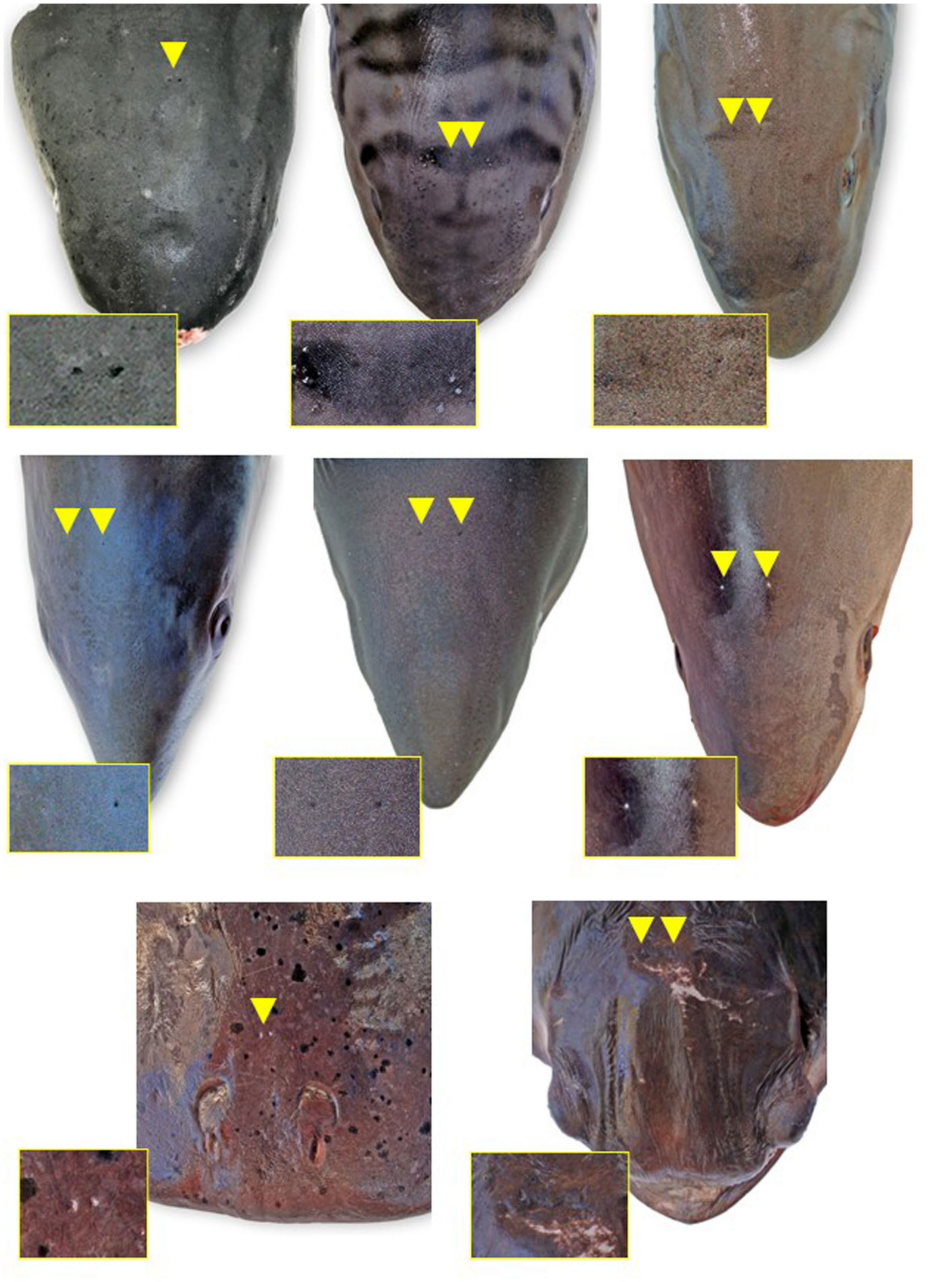
Endolymphatic pores (arrowheads and insets) location and morphology across select elasmobranch species. The pores tend to be located just caudal to the eyes and endolymphatic fossa on either side of midline. **(A)** Broadnose sevengill shark (*Notorynchus cepedianus*), **(B)** leopard shark (*Triakis semifasciata*), **(C)** brown smooth-hound shark (*Mustelus henlei*), **(D)** shortfin mako shark (*Isurus oxyrinchus*) (photo angle distorts bilateral symmetry), **(E)** salmon shark (*Lamna ditropis*), **(F)** common thresher shark (*Alopias vulpinus*), **(G)** Pacific electric ray (*Tetronarce californica*), and **(H)** bat ray (*Myliobatis californica*) (photo angle distorts bilateral symmetry).

**Figure 4:**
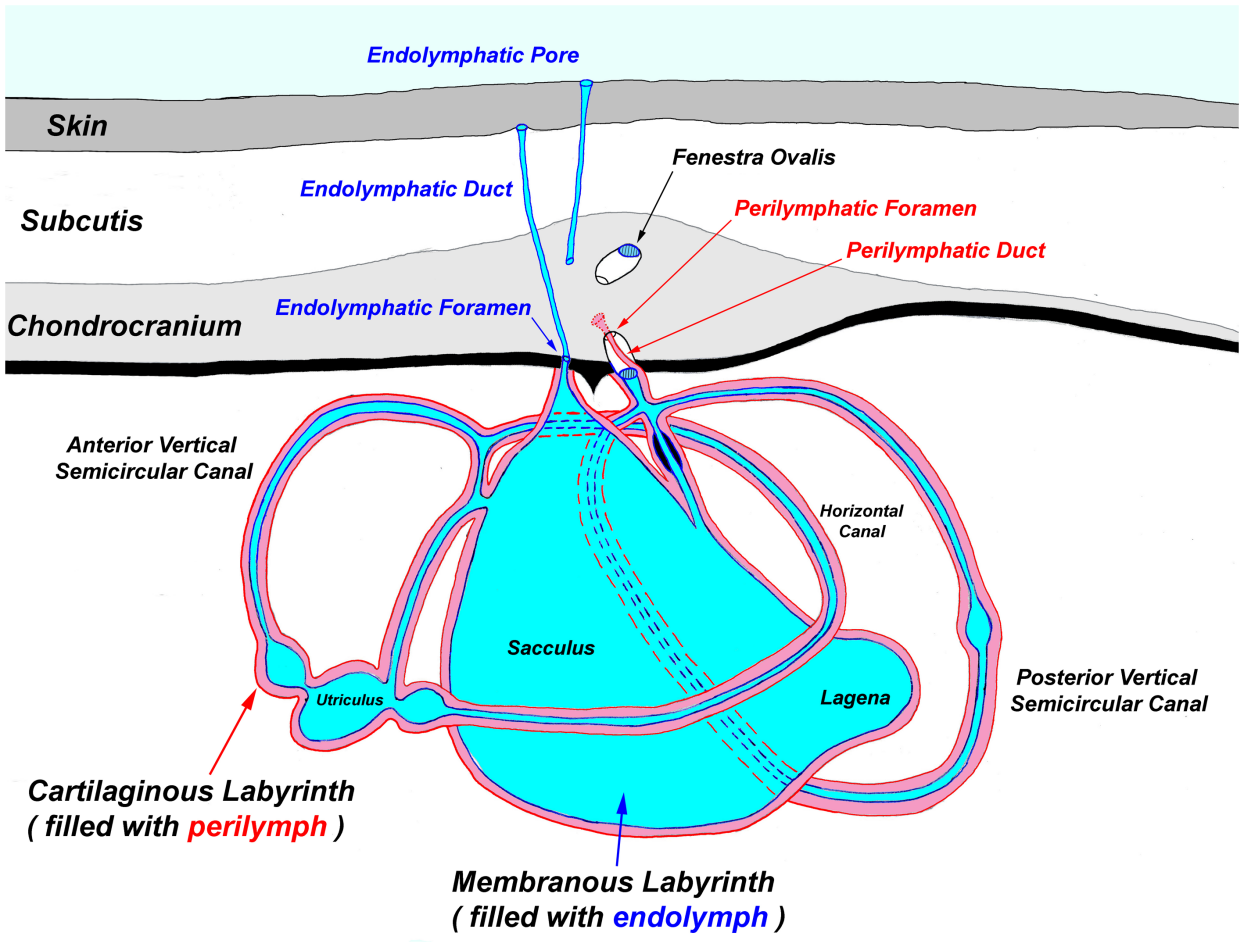
Generalized diagram of the elasmobranch inner ear. Notably, the endolymphatic pores and ducts provide direct entry from the external environment to the inner ear.

Prior to the start of the chondrocranial dissection, it is first necessary to identify key landmarks: nares, eyes, spiracles, Ampullae of Lorenzini, endolymphatic fossa, and endolymphatic skin pores (ELP; Figures 1–3). Focus should be on location of the endolymphatic fossa (Figure 2), which defines the cranial vault, and the ELPs, which are openings for the endolymphatic ducts (ELD; Figures 3, 4) (18).

**Figure 5:**
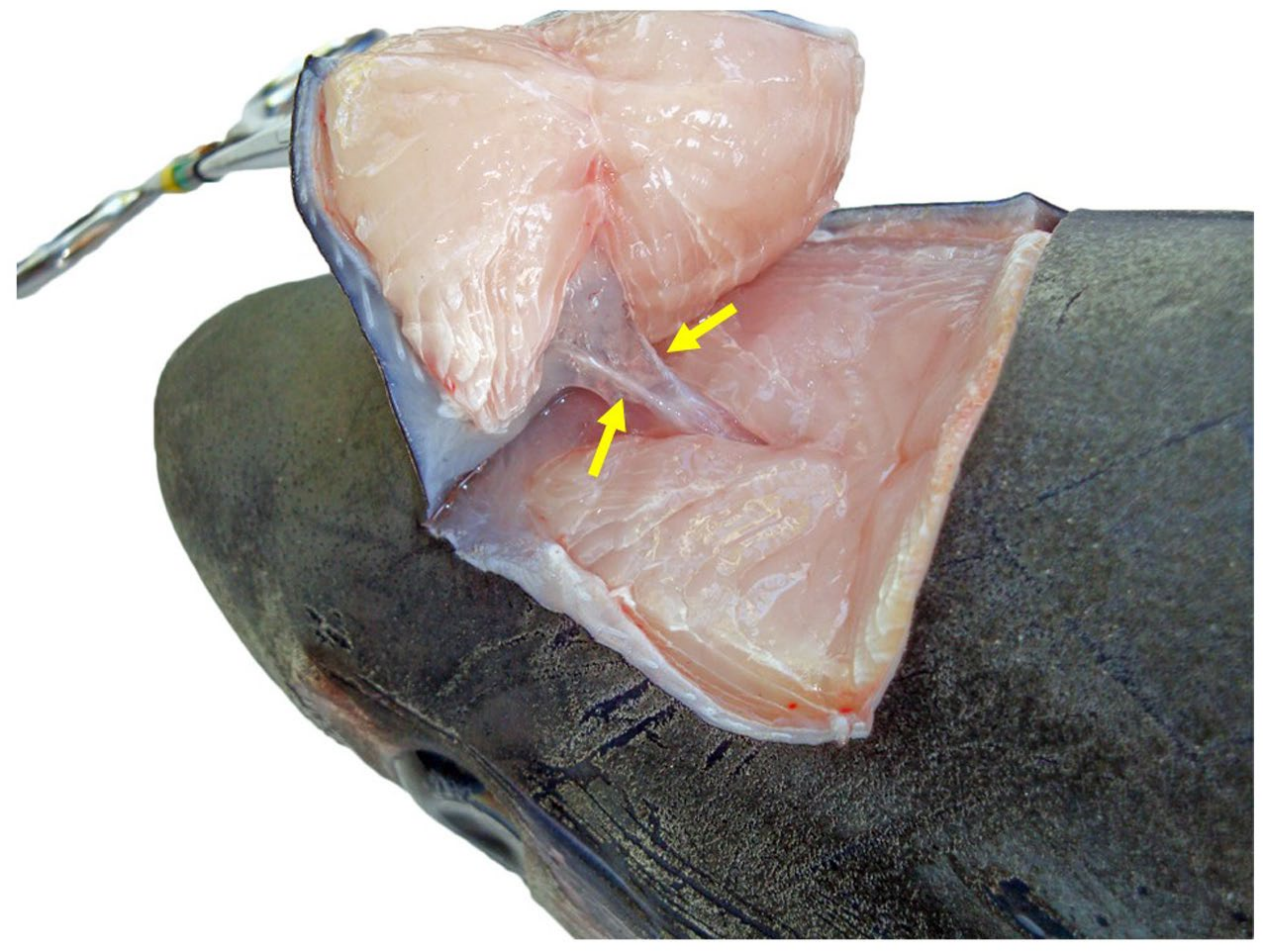
Dorsocaudal view of the paired endolymphatic ducts of a common thresher shark (*Alopias vulpinus*) that connect the outside environment and endolymphatic pores to the sacculus of the inner ears.

The most ergonomic approach is to sit in front of the head facing caudal. Locate the ELPs (Figures 3, 4), which connect to the inner ears via the ELDs (Figures 4, 5). These paired pores sit just off midline, typically caudal to the eyes, and dorsal to the spiracles. Morphology and location vary widely across species, but the ELPs are typically quite small (e.g., <1–2 mm in diameter), especially in sharks (Figure 3) (18). Skin pores for Ampullae of Lorenzini and the cephalic lateral line system are often similar in size and must be distinguished. Identification of ELPs in species with variable skin pigmentation (e.g., leopard sharks, *Triakis semifasciata*) may be difficult or impossible (Figure 3).

If endolymphatic skin pores cannot be visually identified, locate the endolymphatic fossa, a shallow midline oval depression situated dorsally, between the eyes (Figure 2) (18, 19). The endolymphatic fossa is at the apex of the chondrocranium and bounded laterally by the orbits. ELPs are always located at the caudal margin of the fossa. There is no subcutaneous fat or muscle overlying the dorsal aspect of the chondrocranium, so digital pressure can be used to identify the hard margins of the concave endolymphatic fossa. Digital pressure may also cause endolymph with otoconia (crystals in the inner ear that aid in vestibular and auditory sensing) (20, 21) to be expressed from the pores. This will confirm identification of ELPs but does not reliably occur in most species.

Once the ELPs have been identified, the entire dorsal aspect of the head should be cleaned and disinfected using Lysol^™^ or Clorox^™^ wipes, or gauze soaked with 70% isopropyl alcohol. Wipes should be moved rostral to caudal, covering the entire endolymphatic fossa and skin pores with large ~4–6 cm margins. At a minimum, disinfect three times, using a new wipe or gauze pad each time.

**Figure 6:**
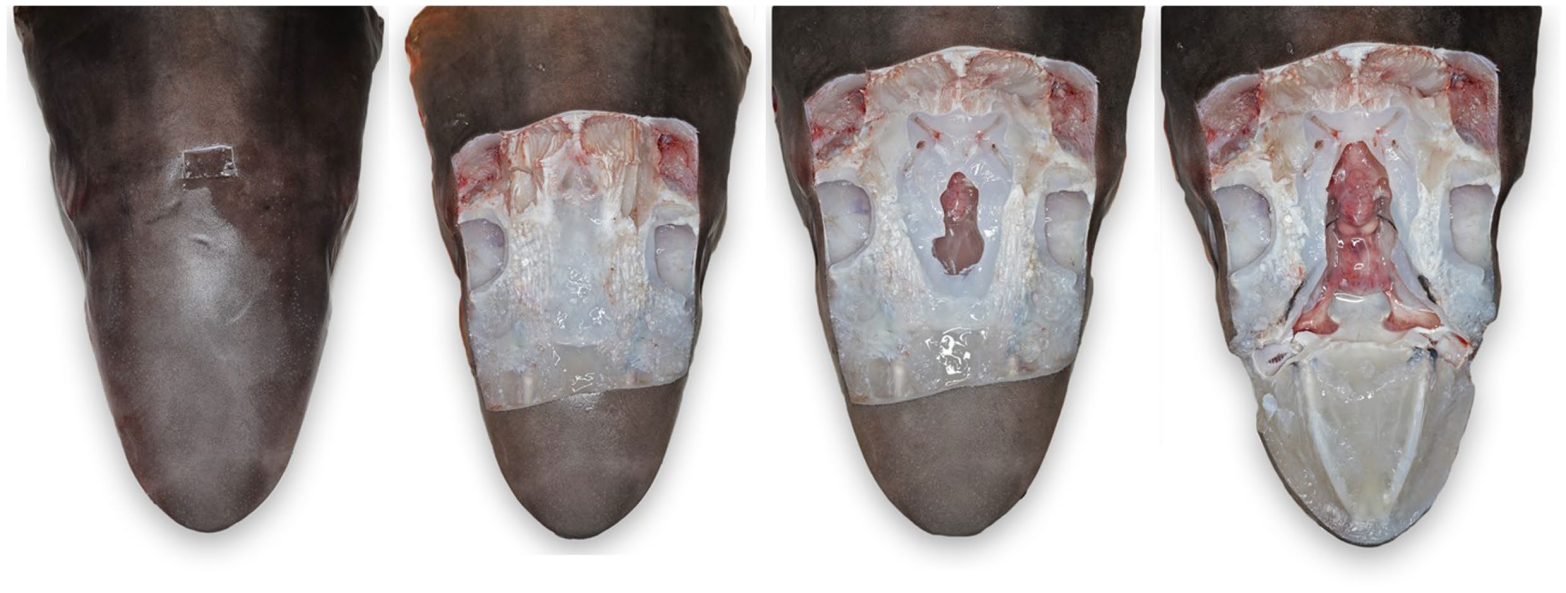
Progressive exposure of chondrocranium and brain vault of a tope shark (*Galeorhinus galeus*). **(A)** Initial incision made around endolymphatic pores to sample the tissue surrounding the endolymphatic ducts. **(B)** Extended exposure of dorsal aspect of the chondrocranium, brain vault is not yet exposed. **(C)** Initial exposure of brain and inner ears for sterile sampling of extradural fluid and inner ear perilymph. **(D)** Complete exposure of main chondrocranial structures, including rostral bars, ampullae of Lorenzini, olfactory capsule, brain, and inner ears.

To maintain an aseptic approach, don new gloves, clean gloves with disinfecting wipes, and use sterile instruments and a new sterile scalpel blade. Make a stab incision lateral to the ELP. Once through the skin, invert the scalpel blade to incise from “inside out” to reduce blade dulling and make a rectangle around the ELPs (~1–2 cm margins around both ELPs) (Figure 6A). Reflect the skin and assess subcutaneous tissues surrounding the endolymphatic ducts (Figure 5). Periductal inflammation is common among stranded sharks; a hemorrhagic subcutis often consistent with trauma. Sample tissues around the ducts for cytology and microbiology. If there is sufficient fluid, collect and freeze for future evaluation. Fix the rectangle of tissue with ELPs and ELDs for histopathology.

Next, extend the skin incisions in all directions to expose the dorsal aspect of the chondrocranium (Figure 6B). The ELPs typically lie directly over the inner ears, which demarcate the caudal aspect of the cranial vault. The goal is to expand the dissection caudally (to the level of the spinal cord) to expose both otic capsules (housing the inner ears) and then laterally and dorsally to expose left and right orbits and midline endolymphatic fossa. The caudal margin of the fossa is characterized by four small foramina (1–3 mm; Figures 2, 4) (18, 19). Connective and/or inflammatory tissue may obscure these openings in the chondrocranium. The smaller rostral pair of round endolymphatic foramina are termination points for the endolymphatic ducts (Figure 4). Ducts are fragile and often severed during the dissection (Figure 5). The larger caudal pair of oval perilymphatic foramina are associated with sound conduction and do not have attached external ducts (Figure 4) (18). Excising the dorsal aspect of the orbits and retrobulbar connective tissue is optional but often simplifies the approach to the brain and inner ears, particularly in smaller animals.

Once the top of the chondrocranium is fully exposed (Figure 6B), repeat the disinfection procedure. Additional disinfection is necessary because of potential microbial contamination from inflamed periductal tissues, and the orbital and branchial cavities. Instruments should be similarly disinfected, a new scalpel blade used, and gloves cleaned or changed. In smaller sharks and rays, the calvarium, including the entire endolymphatic fossa and dorsal aspects of both otic capsules, can often be removed in one piece. Holding the scalpel parallel to the table, make a deep 360-degree coronal incision around the periphery of the fossa and through the caps of both otic capsules. The dissection requires cutting through multiple layers of the robust cartilaginous labyrinth of the inner ears (Figure 4) and may involve significant effort and more than one scalpel blade. With larger sharks, the calvarium and otic capsules may be exposed more gradually by shaving off thin slices of chondrocranium until the cranial vault is open and otic capsules exposed (Figure 6C). Collect samples of the extradural fluid (EDF) overlying the brain for cytology, bacterial, and fungal cultures. Typically, the cranial vault is entered before the inner ears are exposed, but the exact order of sampling is irrelevant. Repeat the aseptic sampling for inner ear perilymph (clinically indistinguishable from endolymph and will be subsequently referred to as perilymph).

After sterile samples have been collected, continue shaving down cartilage to cleanly expose the entire brain, inner ears, and olfactory lamellae (Figure 6D). Use nares as landmarks for locating the olfactory capsule and lamellae and ensure that olfactory bulbs and tracts are exposed. The olfactory tracts and bulbs are often quite deep, diving ventral from the olfactory lobes; patience and precision are needed for this deep dissection. Sample the rostral EDF, adjacent to olfactory lobes, tracts, and bulbs, as inflammatory cells and pathogens often settled during prolonged storage prior to necropsy. Rostral EDF and meninges will typically have more inflammation when pathogens use a nasal route of entry.

**Figure 7:**
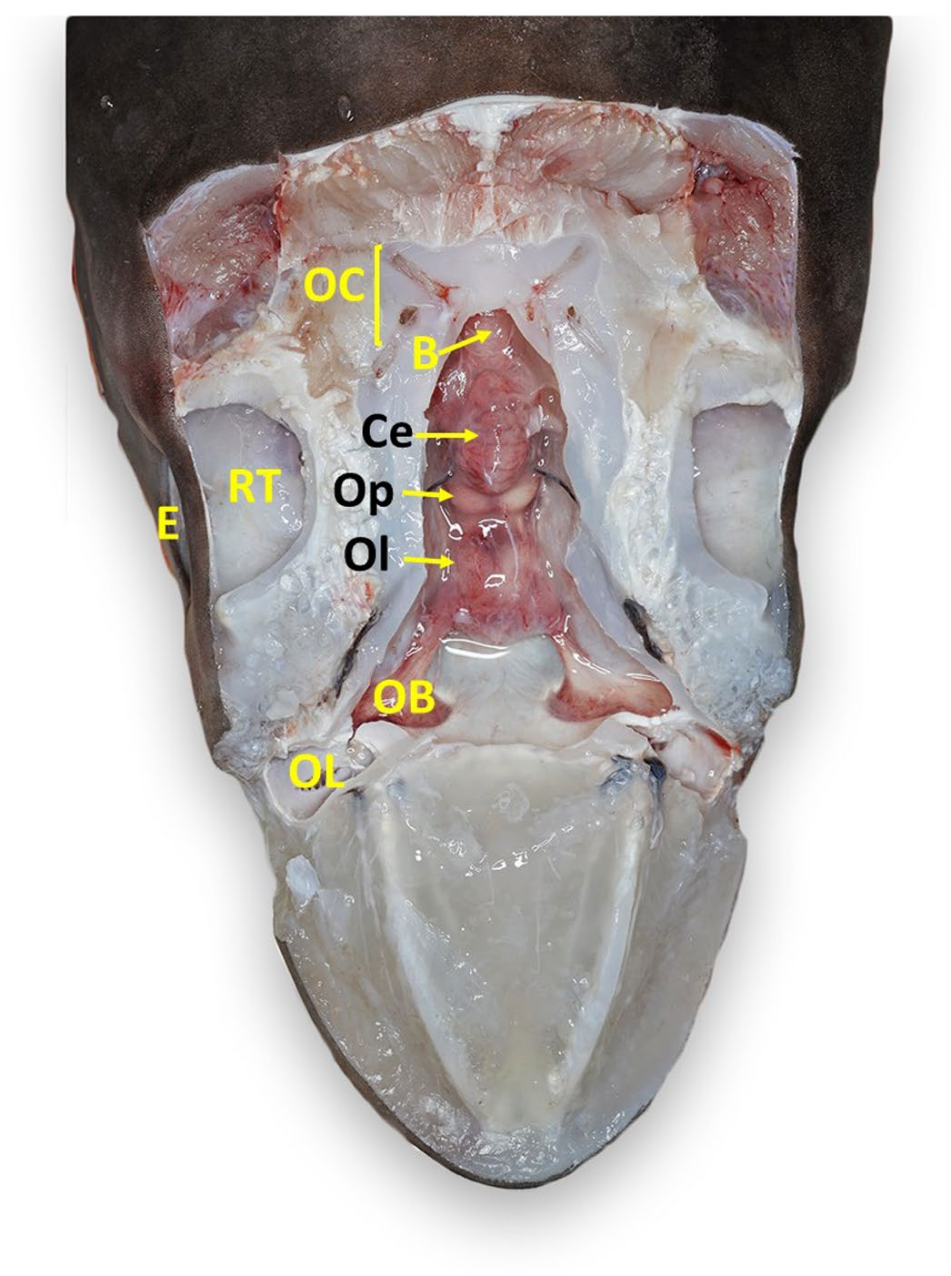
Labeled clinically relevant chondrocranial structures in a tope shark (*Galeorhinus galeus*). OL, olfactory lamellae (right); OB, olfactory bulb (right); Ol, olfactory lobe (fused); Op, optic lobe (fused); Ce, cerebellum; B, brainstem; OC, otic capsule (right); E, eye (right); RT, retrobulbar connective tissue (right).

**Figure 8:**
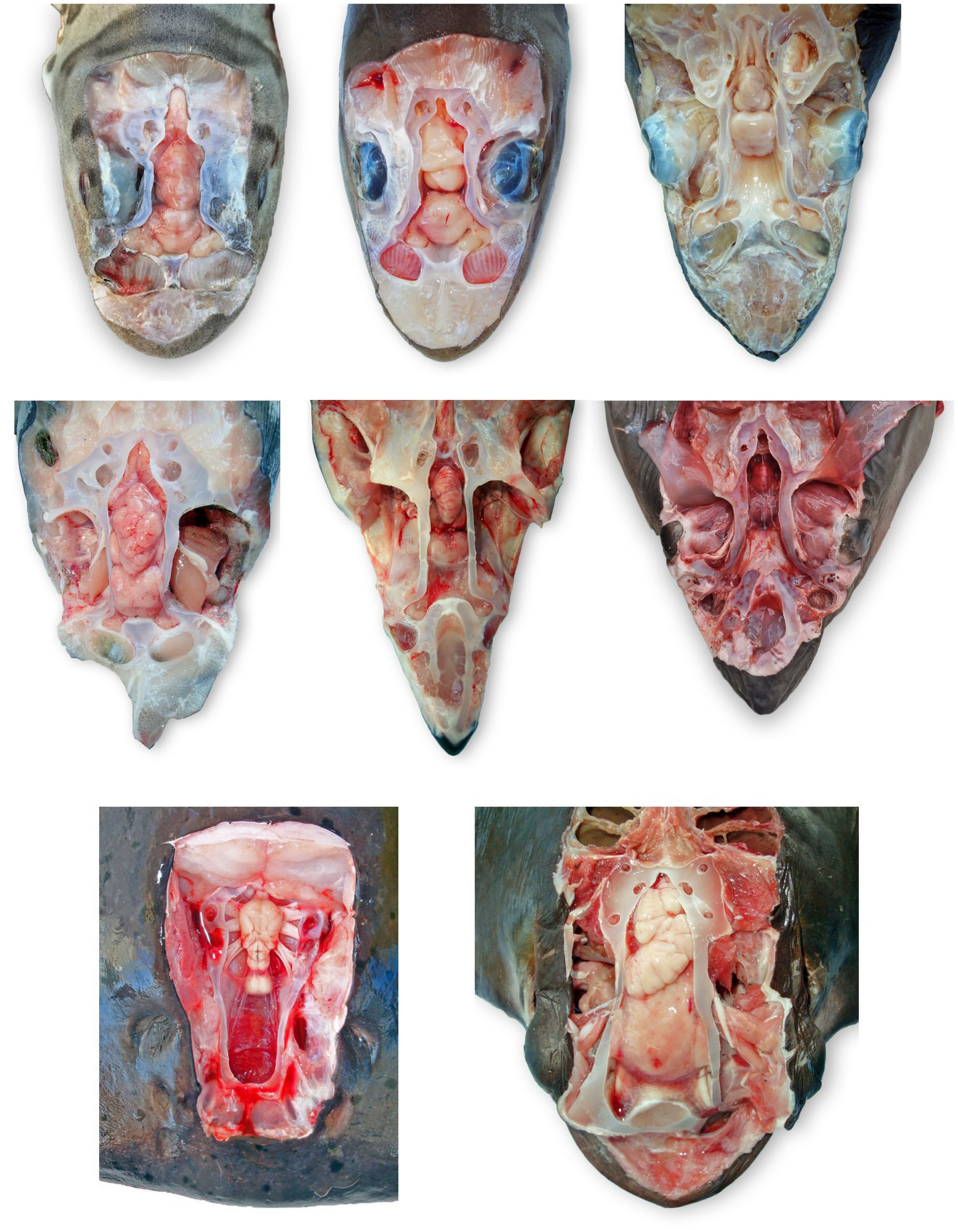
Comparative elasmobranch chondrocraniums with clinically relevant structures exposed; animals have varying degrees of gross pathology. **(A)** Leopard shark (*Triakis semifasciata*), **(B)** brown smooth-hound shark (*Mustelus henlei*), **(C)** blue shark (*Prionace glauca*), **(D)** common thresher shark (*Alopias vulpinus*) with right rostrum and globes removed, **(E)** shortfin mako shark (*Isurus oxyrinchus*) with eyes removed, **(F)** white shark (*Carcharodon carcharias*), **(G)** Pacific electric ray (*Tetronarce californica*) with olfactory bulbs and lamellae not exposed, **(H)** bat ray (*Myliobatis californica*).

**Figure 9:**
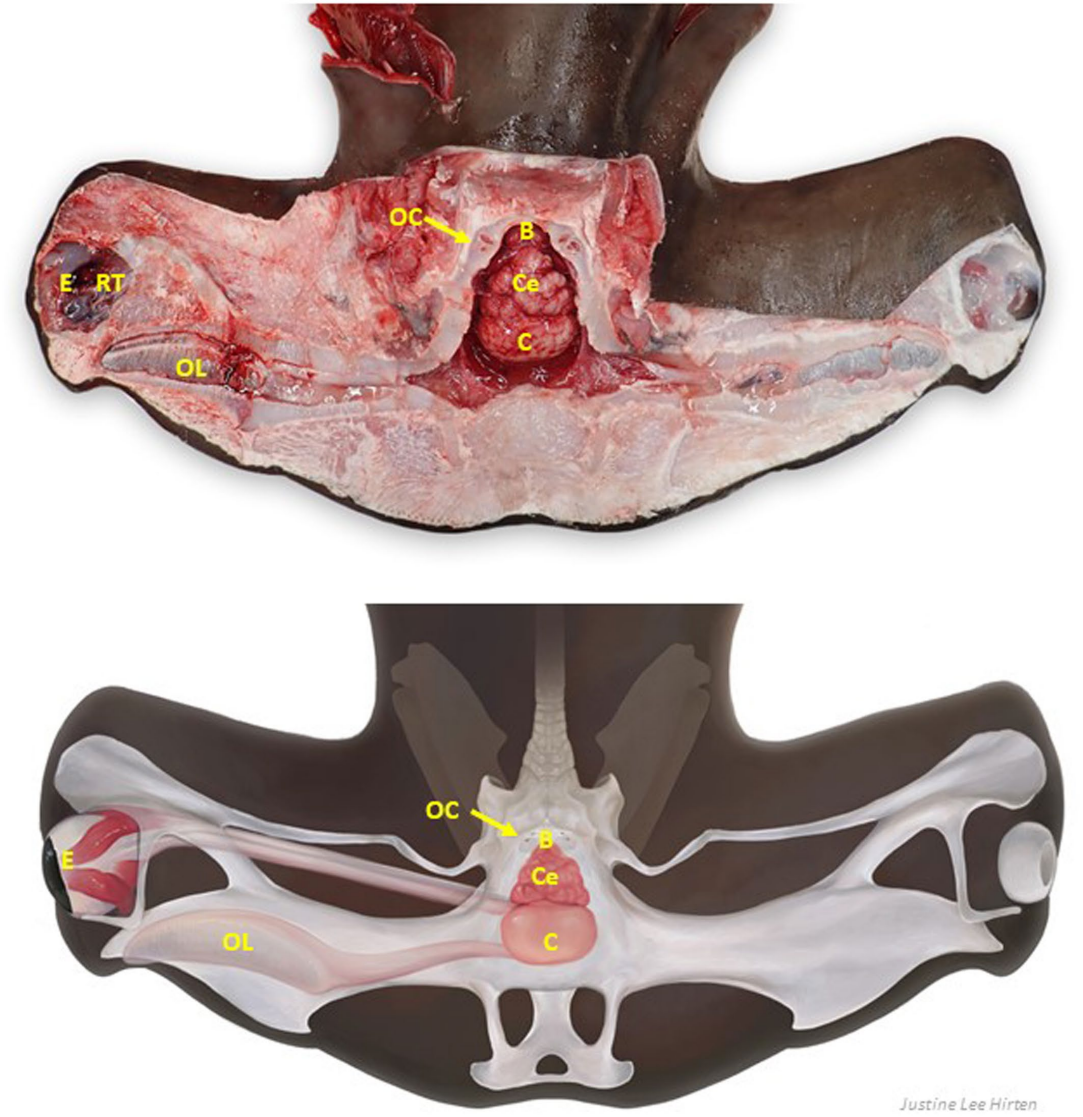
Labeled clinically relevant chondrocranial structures in a scalloped hammerhead shark (*Sphyrna lewini*) **(A)** gross necropsy and **(B)** drawn representation of cartilaginous and key soft tissue structures. OL, olfactory lamellae (right); C, cerebrum (predominately fused olfactory lobes); Ce, cerebellum; B, brainstem; OC, otic capsule (right); E, eye (right); RT, retrobulbar connective tissue (right).

With all relevant structures exposed (Figures 7–9), examine each for pathology. Inner ear perilymph and endolymph should be clear to slightly straw colored; meninges should be thin and clear, and the brain visible through them. Meningeal and parenchymal vasculature should be non-congested; extradural fluid clear to slightly serosanguinous. The parenchyma of the brain should be pale tan and homogeneous, with a smooth surface, and olfactory lamellae a uniform salmon pink to pale red.

### Eyes

After a thorough external ocular exam (assessing conjunctiva, cornea, and third eyelid if present), remove copious gelatinous, retrobulbar connective tissue to expose the six extraocular muscles (22). Evaluate the extraocular muscles for hemorrhage and surrounding tissue for inflammation and hemorrhage. Sever extraocular muscles one-by-one and then apply tension on the globe to expose the optic nerve and optic pedicle. Palpation may be needed to locate and distinguish the optic nerve from the pedicle, a hard cartilaginous extension from the chondrocranium. Cut the optic nerve and optic pedicle to remove the eye. Make a circumferential incision of the globe to evaluate the lens, vitreous and aqueous humor, and retina.

### Thymus

**Figure 10:**
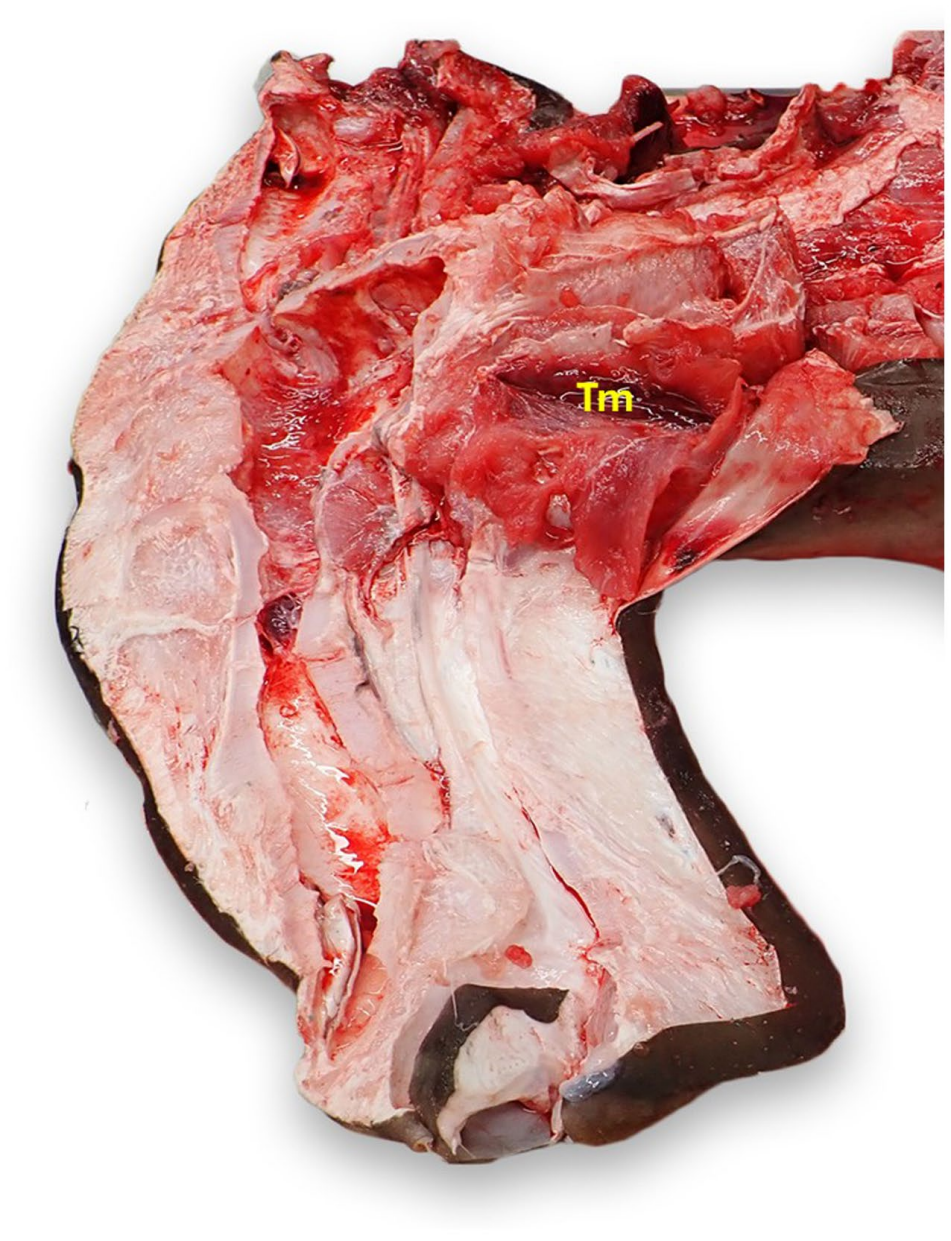
Thymus (Tm) location in a scalloped hammerhead shark (*Sphyrna lewini*) with brain and eyes removed, from a left dorsal view. The thymus is generally bilateral and dorsomedial to the gills, but exact location varies widely by species and age. The thymus in this shark was more cranial than in other species examined.

The thymus is a bilateral, flattened, red-brown tissue that most commonly lies dorsomedial to the first gill slit (Figure 10) (23). Incise the dermis dorsal to the first gill slit. Removal of the dermis in a larger area may be necessary to locate the unencapsulated organ. The thymus may not be readily apparent, and tends to lie deeper in mature animals (23).

### Jaws and oral cavity

Evaluate lips, gingiva, teeth, and oral cavity for trauma or other lesions. This is particularly relevant for wild sharks, as hook injuries are common, especially at the commissures. Note any broken or missing teeth.

### Thyroid

**Figure 11:**
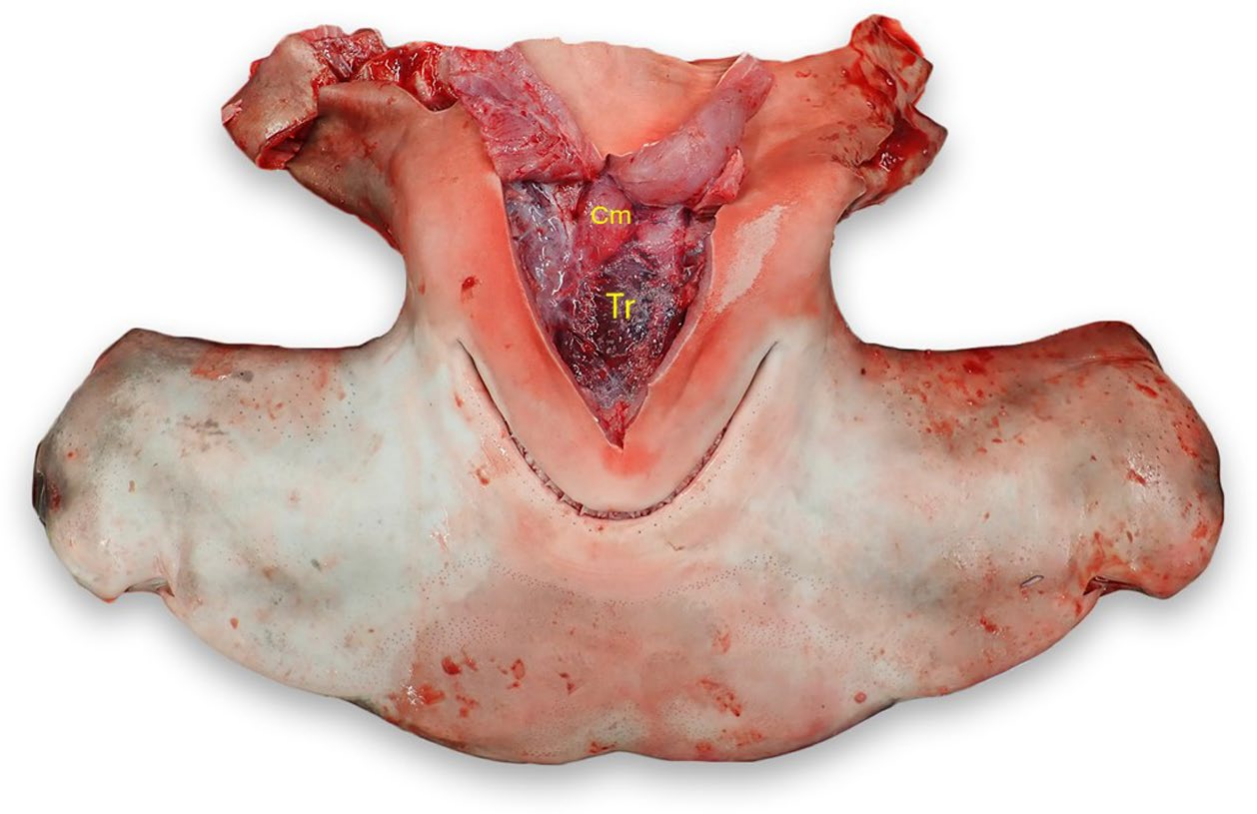
Thyroid (Tr) location in a scalloped hammerhead shark (*Sphyrna lewini*). The thyroid is located in loose connective tissue between the coracohyoid muscles (Cm) dorsally and the coracomandibular and interhyoideus muscles laterally and ventrally (reflected).

With the head in dorsal recumbency, make a stab incision along the medial aspect of Meckel’s cartilage (lower jaw) and follow the cartilage to make a “V” incision along the cartilage. Incise the interhyoideus, intermandibularis, and coracomandibular muscles, which should expose the thyroid if grossly visible. The thyroid is located within the loose connective tissue between the ventral coracohyoid muscles and the dorsal aspect of the coracomandibular muscles (Figure 11) (24, 25).

### Microbiology

Three sites are routinely cultured for bacterial and fungal pathogens: (1) subcutaneous tissues surrounding endolymphatic ducts, (2) EDF and meninges overlying the brain, and (3) inner ear perilymph. Cultures are always done first to minimize microbial contamination. If pursuing in-house culture, streak blood agar and Sab-Dex plates following standard microbiological techniques and maintain at room temperature, checking plates daily for growth (17). Colony morphology (e.g., size, shape, color, margins, hemolysis etc.) should be characterized grossly; cellular composition (i.e., microbial size, shape, and motility) assessed via cytology (17). If there is mixed growth, describe major colony types, estimate abundance, and streak unique colonies onto new plates. Pure growth plates or those with minimal contaminants can be submitted for identification, typically via matrix-assisted laser desorption/ionization-time of flight [MALDI-TOF] and/or 16 s rRNA PCR. Alternatively, Amies agar gel transport swabs (e.g., BD BBL^™^ CultureSwab^™^ Plus) can be inoculated and submitted for culture at appropriate diagnostic labs.

### Cytology

The same three sites cultured for microbial pathogens should also be assessed cytologically. Primary fluid samples include (1) subcutaneous fluid around ELDs, (2) EDF overlying the brain, and (3) inner ear perilymph. As stated above, it is often advantageous to take multiple EDF samples (i.e., an initial dorsal aspirate of fluid overlying the cerebellum and a second rostral/ventral sample from the base of the olfactory lobes). Optional cytology samples include the perichondrial surface of the calvarium (often holding inflammatory exudate), inner ear otoconia from the sacculus (to characterize normal otoconia), and meninges (which may hold larger pathogens, like nematodes).

Fluid cytology aspirates are taken with transfer pipettes or syringes, with or without needles. Thicker exudates are sampled via scraping with a spatula or dull scalpel; tissue samples (e.g., meninges) taken with scissors. Wet mount cytology samples should be mounted onto glass slides and then coverslipped for dark-field exam. Fluid and tissue samples should be thin enough to allow identification of inflammatory cell types and unicellular pathogens. Thicker exudates, and microbial colonies from plate agar, can be diluted with saline, prior to coverslipping, to allow for separation of cellular components and easier identification. Allowing clear fluids to settle in cryovials for 10–15 min, prior to sampling, can help maximize detection of pathogens and inflammatory cells.

If dark-field microscopy is unavailable, wet mount preparations can still be assessed by lowering the microscope condenser, closing the condenser aperture, and decreasing available light until cellular detail is discerned. Phase contrast can also be used to help visualize cells and pathogens if dark-field is not available. Wet mount exams allow for immediate assessment of inflammatory response and presence of etiologic agents, especially motile pathogens (e.g., motile bacteria, ciliated or flagellated protozoa).

Cytology preparations (e.g., push or pull smears, tissue imprints, and squash preps) can also be air-dried and stained to provide additional diagnostic material using standard (26). After a small drop is placed on one end of a glass slide, a second slide is used at a 45-degree angle to “push” and spread the fluid over the slide. “Pull smears” are made by placing a fluid sample between two slides and pulling in opposite directions. Tissue imprints are made by gently blotting a tissue dry, then lightly touching tissue samples onto a glass slide multiple times. Squash preps are made by crushing a tissue sample between two slides.

Slides should be thoroughly air-dried or lightly heated prior to alcohol fixation and staining. Use of modified Wright-Giemsa stains allows for greater cell identification, clearer visualization of bacteria and fungi, and creates a durable record if stained slides are dried and coverslipped with permanent mounting media (e.g., Permount^®^). Stained, coverslipped cytology slides can also be examined with oil immersion objectives (1,000X magnification), increasing diagnostic accuracy.

### Histology

For histological sampling of smaller sharks and rays, the entire brain, pituitary gland, inner ears, and olfactory lamellae should be immersion fixed in 10% formalin, along with the ventral aspect of the chondrocranium. Fixing the brain whole, with inclusion of supporting chondrocranial elements (i.e., olfactory capsules, palate, pituitary fossa, and otic capsules), requires extensive dissection, often with a heavy knife, but minimizes excisional damage. This method is also ideal for those animals with friable brains due to autolysis. All tissues should be fixed with at least a 10:1 formalin to tissue ratio.

For larger elasmobranchs, the brain should be separated from the chondrocranium prior to histologic fixation. The decision on whether to excise the brain with or without olfactory bulbs is dependent on cerebral anatomy and size. In species with short, thick olfactory tracts (e.g., Triakidae houndsharks), it is best to keep the olfactory bulbs with the rest of the brain. In contrast, animals with long thin olfactory tracts (e.g., white sharks [*Carcharodon carcharias*] and broadnose sevengill sharks [*Notorynchus cepedianus*]), the preferred method is to sever the olfactory tracts, prior to removal of the brain, and leave the olfactory bulbs with the olfactory lamellae (Figure 8).

To remove brains with attached olfactory bulbs, first separate the olfactory bulbs from adjacent lamellae using a scalpel to cut short olfactory nerves bridging the two organs (27). Olfactory nerves are not grossly visible, so incisions are made blindly, leaving the entire olfactory bulb attached to the tract. Once left and right olfactory bulbs are free, the cervical spinal cord can be transected distal to the brainstem. Holding the spinal cord with forceps, and/or using a tissue spatula to support the brain, elevate the brain dorsally to expose cranial nerves. Use scissors or a scalpel to transect cranial nerves close to the chondrocranial cartilage. Inverting the head, or turning it to the side, will allow gravity to expose cranial nerves and ease removal of the brain.

For brains where olfactory tracts are cut and olfactory bulbs left attached to the lamellae, the procedure is essentially the same. The only difference is that olfactory bulbs need to be excised separate from the rest of the brain. In this scenario, olfactory bulbs are excised together with attached olfactory lamellae. A scalpel is used to cut through the olfactory capsule, separating it from the rest of the chondrocranium. The brain, olfactory bulb and lamellae are then fixed in formalin. Large brains may benefit from injecting 10% formalin into the parenchyma or sectioning lobes to expose ventricles and improve fixation (7).

Following removal of the brain, the inner ears and olfactory lamellae can be sampled for histology. Inner ears (semicircular canals and membranous sacs) are sampled using sharp dissection to excise otic capsules (cartilaginous and membranous labyrinths combined) using deep vertical cuts to chop out rough cube-shaped sections of the chondrocranium. In similar fashion, olfactory lamellae are removed via carving out the entire olfactory capsule prior to immersion fixation in formalin.

### Trimming tissues for histopathology

Fixed tissues can be trimmed into histology cassettes 48 h after immersion in 10% formalin. Cut tissues into 2–5 mm thick pieces with a sharp, single-edged razor blade following standard protocols for tissue trimming (28, 29). Orient tissues with the side of interest face down in the cassette. Tissues of similar consistency may be included within the same cassette but avoid overcrowding. Small or friable samples should be sandwiched between tissue sponges to prevent loss. Mineralized tissues, such as the endolymphatic pores with surrounding dermal denticles, may require decalcification prior to sectioning.

**Figure 12:**
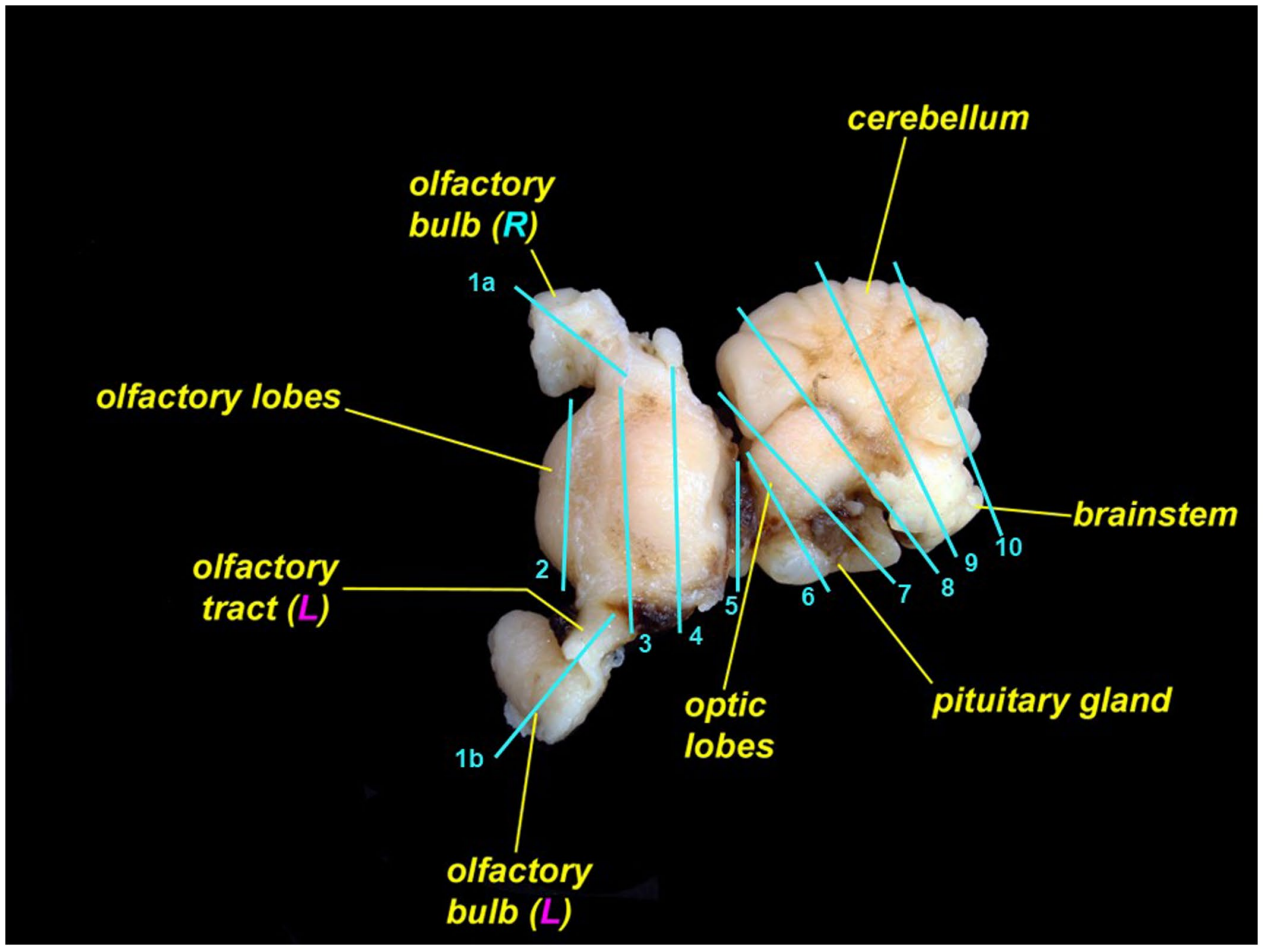
Leopard shark (*Triakis semifasciata*) brain with suggested locations to trim for histopathologic evaluation. The rostral brain (rostral to cut 5) is pictured from the dorsal aspect; the caudal brain (caudal to cut 5) is rotated and is pictured from the lateral aspect.

Skin containing endolymphatic pores and ducts should be trimmed adjacent to one or both pores. Cutting skin with the dermis and subcutis face up will reduce artifacts. To section the brain, we recommend including a sagittal section of each olfactory bulb and olfactory tract (Figure 12, cut 1a and 1b); rostral telencephalon (Figure 12, cut 2); mid telencephalon (Figure 12, cut 3); caudal telencephalon (Figure 12, cut 4); diencephalon and optic chiasm (Figure 12, cut 5); optic lobe and pituitary gland (Figure 12, cut 6); rostral cerebellum, mid optic lobe, and pituitary gland (Figure 12, cut 7); cerebellum, caudal optic lobe, and fourth ventricle (Figure 12, cut 8); cerebellum and brainstem (Figure 12, cut 9); and caudal cerebellum and brainstem (Figure 12, cut 10) (27, 30). Exact sections will vary with different brain morphology.

If olfactory lamellae were fixed together with olfactory bulbs, use sagittal cuts so that trimmed pieces include both organs. If lamellae are fixed separately, either sagittal or transverse sections will work. Inner ear samples should be trimmed to include the sacculus and surrounding otic cartilage. Tissue sponges may be needed to prevent loss of inner ear samples during processing.

### Artifact interpretation

Elasmobranchs, particularly stranded animals, often have some degree of autolysis and/or scavenging damage. Both can complicate interpretation of necropsy findings. Scavenging of moribund and dead sharks and rays most commonly affects the gills, globes, and cloaca. Despite scavenging, the chondrocranium, brain, and inner ears are often intact.

Autolysis (i.e., postmortem degradation associated with endogenous and exogenous bacteria, as well has host enzymes and bile) is another common artifact, particularly in stranded and warm water elasmobranchs that are not collected for many hours after death. Autolysis of the brain manifests as tissue softening, loss of integrity, and eventual liquefaction. If cytology and/or culture from an autolyzed brain reveal heavy, mixed bacterial growth, then postmortem bacterial overgrowth can be assumed, which can preclude a definitive diagnosis (31, 32).

## Results

### Relevant anatomy

All elasmobranch species examined had identifiable ELPs, although they were larger and easier to locate in rays versus sharks (Figure 3). ELPs were often pinpoint and difficult to find in small sharks, particularly tope and leopard. When skin pores could not be readily found, the palpable median endolymphatic fossa was a reliable landmark, providing an initiation point for the dissection and exposure of paired ELDs (Figure 2). Unique to elasmobranchs, these ducts are often overlooked by mammalian and teleost pathologists. Tracing ELDs and careful exposure of the dorsal chondrocranium provided access to both inner ears and brain.

Chondrocranial anatomy was conserved between species, with the endolymphatic fossa defining the apex of the calvarium, and paired perilymphatic and endolymphatic foramina defining the dorsal aspect of the otic capsules. Brain morphology was generally consistent, with all examined shark and ray species having large cerebellums, a relatively small optic tectum, and large fused olfactory lobes (i.e., telencephalon/cerebrum; Figures 7, 8) (33). The primary difference between shark species was with the arrangement of olfactory tracts and bulbs. In broadnose sevengill and white sharks, olfactory tracts were long and thin, with olfactory bulbs located at considerable distance from the rest of the brain. Hound sharks (i.e., leopard, brown smooth-hound [*Mustelus henlei*], and gray smooth-hound [*Mustelus californicus*]), in contrast, had short robust olfactory tracts and olfactory bulbs near olfactory lobes. Differing central nervous system anatomy dictated slightly different approaches to histological sampling.

### Causes of mortality

The described chondrocranial approach as the first step of a whole-body dissection was implemented in the necropsy of 189 elasmobranchs, representing 18 shark and ray species, over a 13 year period from 2011 through 2023 (Table 1). Included were necropsies of 156 wild stranded animals, 12 under managed care at five California institutions, and 21 caught by fisherpeople. Meningoencephalitis was found in 70% (118/168) of stranded and aquarium-housed elasmobranchs and was the most common cause of mortality in this study (Table 1). The vast majority of meningoencephalitis cases were due to bacteria (*Carnobacterium maltaromaticum*) or protozoa (*Miamiensis avidus*) (Chang and Okihiro, manuscript in preparation).

**Table 1:** Primary causes of mortality in stranded wild and aquarium-housed elasmobranchs necropsied from 2011–2023.

Species	Total necropsy	Meningo-encephalitis	Trauma	Other	Healthy (caught)	Etiology not determined
Leopard Shark *Triakis semifasciata*	67	48	5	1	6	7
Salmon Shark *Lamna ditropis*	40	37	0	0	1	2
Common Thresher Shark *Alopias vulpinus*	18	12	5	0	0	1
White Shark *Carcharodon carcharias*	9	1	7	1	0	0
Tope Shark *Galeorhinus galeus*	9	6	0	2*	0	1
Bat Ray *Myliobatis californica*	7	4	1	0	0	2
Broadnose Sevengill Shark *Notorynchus cepedianus*	7	2	0	5	0	0
Shortfin Mako Shark *Isurus oxyrinchus*	6	4	0	0	1	1
Blue Shark *Prionace glauca*	6	0	3	0	3	0
Grey Smooth-hound Shark *Mustelus californicus*	5	0	0	0	5	0
Shovelnose Guitarfish *Rhinobatos productus*	4	1	0	0	3	0
Round Stingray *Urobatis halleri*	3	0	0	1	2	0
Pacific Electric Ray *Tetronarce californica*	3	1	0	2	0	0
Brown Smooth-hound Shark *Mustelus henlei*	1	1	0	0	0	0
Prickly Shark *Echinorhinus cookei*	1	0	0	1	0	0
Thornback Guitarfish *Platyrhinoidis triseriata*	1	0	1	0	0	0
Scalloped Hammerhead Shark *Sphyrna lewini*	1	0	1	0	0	0
Pacific Angel Shark *Squatina californica*	1	1	0	0	0	0
TOTAL	189	118	24	12	21	14

Included are necropsy data from some healthy elasmobranchs caught by fisherpeople. *Primary cause of mortality was capture/transport stress, but an underlying meningoencephalitis and otitis interna was considered contributory.

*Carnobacterium maltaromaticum* meningoencephalitis occurred in salmon sharks (*Lamna ditropis*), shortfin mako sharks (*Isurus oxyrinchus*), common thresher sharks (*Alopias vulpinus*), and one Pacific electric ray (*Tetronarce californica*). *Carnobacterium maltaromaticum* was consistently identified via cytology and bacteriology (culture on blood agar followed by identification with PCR and/or MALDI-TOF) in subcutaneous tissues around ELDs, inner ear perilymph, and EDF. The pathogen was extremely hardy and could be isolated from animals that had been frozen for months or years.

*Miamiensis avidus*, a scuticociliate parasite, was the most common cause of protozoal meningoencephalitis in stranded elasmobranchs. *Miamiensis avidus* meningoencephalitis was found in 48 leopard sharks, 4 bat rays (*Myliobatis californica*), one common thresher shark, and one brown smooth-hound shark. *Miamiensis* infections were typically characterized by mixed inflammation of rostral EDF, without visualization of motile protozoa because stranded animals were usually dead for 1–3 days prior to necropsy. Except for the thresher shark infection, all *M. avidus* cases occurred in and around San Francisco Bay, CA. Protozoal infection was confirmed via histology and/or PCR in many instances but was also a presumptive diagnosis based on cytology during large epizootics. Less common types of meningoencephalitis in stranded sharks and rays were verminous, fungal, and streptococcal (Chang and Okihiro, manuscript in preparation).

Excluding gill net by-catch and those intentionally caught via hook-and-line or spear, trauma, most often anthropogenic, was the second most common cause of mortality among stranded sharks and rays, and responsible for 14% (24/168) of reported deaths (Table 1). Types of traumatic injuries included: (1) crush and propeller wounds from boat strikes; (2) abrasions, lacerations, and internal hemorrhage from commercial nets; (3) spinal fracture and hemorrhagic puncture wounds from gunfire; (4) tooth loss, jaw damage, gastric perforation, and deep fin lacerations from fishing hooks and line; and (5) collision from an enclosure-mate. Trauma was the cause of stranding and mortality in white sharks, blue sharks (*Prionace glauca*), thornback guitarfish (*Platyrhinoidis triseriata*), and common thresher sharks.

Among elasmobranchs under human care, causes of death included meningoencephalitis (*M. avidus* and nematodes), granulomatous nephritis, enclosure-mate trauma, and capture and transport stress with chronic meningitis of unknown etiology (Chang and Okihiro, manuscript in preparation).

### Pathogenesis of chondrocranial infections

The location and severity of inflammation observed via cytology reliably suggested the route of pathogen entry and pathogenesis of infections in many animals and was supported by microbiologic and histologic findings. With etiologic agents likely gaining entry via ELPs and ELDs (e.g., *Carnobacterium maltaromaticum*), inflammation and bacteria were present in subcutaneous tissue around ELDs, inner ear perilymph, and EDF overlying the brain (Figures 4, 5). In contrast, pathogens entering via the nares and olfactory lamellae [e.g., *Miamiensis avidus*; (6, 34)] tended to cause the most significant inflammation in rostral meninges and EDF surrounding the olfactory bulbs, with little or no inflammation of subcutaneous tissues surrounding ELDs or inner ear. Sharks and rays without significant nasal, endolymphatic, or inner ear inflammation, are presumed to have been infected via hematogenous spread, or possibly by a direct route of entry (e.g., penetrating foreign body).

## Discussion

This approach to necropsy of the elasmobranch chondrocranium was developed and refined over a 13 year period and has proven applicable to a wide variety of elasmobranch species. In contrast to postmortem protocols which focus on the coelomic and branchial cavities, leaving the head exam to last, we have found that reversing the order of investigation to significantly increase the odds of making a definitive diagnosis, identifying pathogens, and determining pathogenesis. Although the brain does not necessarily autolyze faster than other tissues (35), hours can make the difference between interpretable cytology and histology and post-mortem overgrowth and autolysis (36). Prioritization of the head, together with knowledge of unique features of elasmobranch cranial anatomy, will allow cause of death and likely route of pathogenesis to be determined in more wild elasmobranchs and those under human care. Without an in-depth, methodical approach to the head necropsy, the endolymphatic route of pathogenesis for *C. maltaromaticum* and the olfactory route for *M. avidus* would have been missed.

Although there are distinct anatomic differences between many elasmobranch species (Figures 8, 9), particularly across different superorders, the described protocol utilizes conserved cranial landmarks and can be applied to most elasmobranchs. Awareness of unique elasmobranch anatomy minimizes the chances of overlooking significant lesions and potential routes of pathogen entry into the chondrocranium. The underlying problem is that no formal training is available for elasmobranch necropsy. Although the importance of assessing the central nervous system is well established among mammalian and teleost fish pathologists, only a small percentage will extend dissection and sampling to include the ELDs, inner ears, olfactory bulbs, and olfactory lamellae when working with sharks and rays. Limiting cranial examination to just the brain also limits understanding of pathogen entry and pathogenesis, which are major shortcomings in published reports of infectious meningoencephalitis in elasmobranchs (8–11, 13, 15).

Paired ELPs and ELDs are one of many unique anatomic features of elasmobranchs. Fully developed during late gestation and patent throughout the life of sharks and rays (37, 38), ELPs and ELDs provide a direct route for microbial entry into the otic capsules (18, 39, 40). Once established in the inner ear, pathogens are largely shielded from host immune surveillance and can readily spread to the adjacent cranial vault, meninges, and brain. *Carnobacterium maltaromaticum*, the most common cause of bacterial meningoencephalitis in stranded California elasmobranchs (Chang and Okihiro, manuscript in preparation), almost certainly uses an endolymphatic route of entry based on our findings.

Necropsy of the elasmobranch head should always begin with an aseptic approach to the ELPs and ELDs, with continued use of sterile technique until all three primary sites of potential infection—periductal subcutaneous tissue, inner ear perilymph, and EDF overlying the brain—have been sampled. The assumption is that these three sites are infected until proven otherwise. We encourage the use of as many ancillary diagnostic techniques as possible to identify pathogens and characterize the inflammatory response. The layered use of multiple diagnostic tools—cytology, microbiology, histology, and molecular biology—increases the odds of making a definitive diagnosis.

Cytology and microbiology are two inexpensive, rapid diagnostic highly useful for elasmobranch necropsy. Cytological exam of wet mount and stained preparations provide immediate feedback on inflammation, pathogen presence, and disease pathogenesis, factors which could influence or dictate quarantine and treatment options if animals under managed care share their systems with other animals. Critical decisions on whether to isolate (and treat) or euthanize animals often hinge on pathogen identification because infectivity, speed and mode of replication, as well as virulence, vary widely. Discovery of verminous meningitis in a tope shark carries with it markedly fewer consequences than uncovering necrotizing protozoal encephalitis in a leopard shark. Verminous meningitis is often a chronic, slowly developing disease which may be species specific and treatable; while invasive scuticociliates are highly virulent, lethal, nearly untreatable pathogens with little to no host specificity (41–43). Nematodes have a complex life cycle requiring an intermediate host (44), while scuticociliates are water-borne, highly transmissible, and can multiply via binary fission (45, 46).

*Miamiensis avidus* is a prolific killer of elasmobranchs and teleost fishes and is responsible for repeated epizootics of leopard shark stranding in San Francisco Bay (6) (Okihiro and Chang, manuscript in preparation). In the elasmobranchs included in this study, *M. avidus* was the most common cause of leopard shark strandings. It can, however, be surprisingly difficult to identify with cytology and histology because protozoal numbers are often small, parasites autolyzed quickly, and motility lost with delays associated with slow recovery of stranded animals. Problems with visual detection can also arise because protozoa use a nasal route of entry and involvement may be restricted to just olfactory lamellae and bulbs.

Definitive evidence for *Miamiensis* involvement in the 2017 epizootic only occurred when EDF samples were assessed via mNGS and PCR in 2017 (6). Parasites were subsequently found in tissue sections, but only after extensive histological sampling. This emphasizes the benefits of a layered approach and archival storage of histology samples (in formalin and paraffin blocks) and molecular biology samples (via freezing).

In summary, prioritization of the head during the elasmobranch necropsy, with knowledge of unique anatomy and emphasis on an aseptic approach to dissection and sampling of the endolymphatic system, inner ears, and brain, will greatly increase the odds of making definitive diagnoses, identifying pathogens, and characterizing pathogenesis. Knowledge gained will assist with monitoring the health of wild stocks, as well as providing valuable information to those involved with making decisions for animals under human care.

## Data availability statement

The datasets presented in this article are not readily available because expanded mortality data will be included in a subsequent publication. Requests to access the datasets should be directed to RC rchang@mbayaq.org.

## Ethics statement

Ethical approval was not required for the study involving animals in accordance with the local legislation and institutional requirements because data were collected opportunistically during routine diagnostic submissions.

## Author contributions

RC: Conceptualization, Data curation, Investigation, Methodology, Visualization, Writing – original draft, Writing – review & editing. MO: Conceptualization, Data curation, Investigation, Methodology, Visualization, Writing – original draft, Writing – review & editing.
